# Translational Progress and Clinical Challenges in Bioengineered Bone and Joint Repair

**DOI:** 10.3390/biomedicines14061374

**Published:** 2026-06-18

**Authors:** Anoop Sunkara, Connor Primo McCloskey, David Antonio Dias, Siddhartha Kalala, Jack Thomas Peterson, Maxwell James Latshaw, Arun Kiran Movva, Albert Thomas Anastasio

**Affiliations:** 1 Feinberg School of Medicine, Northwestern University, Chicago, IL 60611, USA; anoop.sunkara@northwestern.edu (A.S.); connor.mccloskey@northwestern.edu (C.P.M.); arun.movva@northwestern.edu (A.K.M.); 2 Rush University Medical College, Rush University, Chicago, IL 60612, USA; 3 Department of Biological Sciences, Washington University in St. Louis, St. Louis, MO 63130, USA; 4 Department of Orthopaedic Surgery, Wake Forest University School of Medicine, Winston-Salem, NC 27157, USA

**Keywords:** musculoskeletal regeneration, tissue engineering, regenerative biomaterials, bone and joint repair, stem cell therapy, orthopedic bioengineering

## Abstract

Musculoskeletal disorders involving bone, cartilage, tendon, and joint tissues represent a leading cause of disability worldwide, and conventional surgical and graft-based interventions are limited by donor site morbidity, incomplete integration, and finite durability. Despite substantial preclinical progress, translation into reliable clinical benefit has remained inconsistent. This narrative review synthesizes recent advances in bioengineered approaches to bone and joint repair, emphasizing how materials design and regenerative strategy selection influence translational feasibility. Advances in scaffold-based systems highlight the role of material composition, architectural organization, and structure–function matching in supporting musculoskeletal regeneration. Regenerative platforms including stem cell therapies, extracellular matrix-derived constructs, and smart materials are evaluated for biological performance, manufacturability, and regulatory feasibility. Early translational and clinical studies demonstrate encouraging outcomes across selected musculoskeletal indications; however, variability in efficacy and adoption highlights persistent barriers to broader implementation. Key challenges include scalable manufacturing, cost and reimbursement uncertainty, and heterogeneity in clinical infrastructure, factors that may also influence access to advanced regenerative therapies. Future innovations should emphasize manufacturability and real-world evidence generation that align with practical clinical pathways.

## 1. Introduction

### 1.1. Global Burden of Musculoskeletal Disease

Musculoskeletal (MSK) conditions are the leading cause of disability affecting an estimated 1.71 billion people worldwide [1]. Beyond related deaths, these conditions contribute to a substantial portion of global disability adjusted life years (DALYs). From 1990 to 2019, the rates of incident cases, deaths, and DALYs have increased by 59.86%, 116.02%, and 77.39%, respectively [2].

Several diseases depict the vast epidemiological scale of MSK conditions. Osteoarthritis (OA) alone affects over 607 million individuals globally, with prevalence continuing to rise [3]. While knee and hip OA are primary contributors, ankle and foot OA remain historically underrecognized in epidemiological datasets. Overlapping with chronic conditions such as OA is the substantial burden of acute MSK trauma. According to the 2019 Global Burden of Diseases, Injuries, and Risk Factors Study (GBD) framework, approximately 178 million new bone fractures occur worldwide each year [4]. A persistent surgical challenge in this population is impaired healing; delayed union or nonunion affects 5% to 10% of long-bone fractures, although this statistic can vary widely by fracture location [5]. Soft tissue injuries compound this burden, with rotator cuff tears present in approximately 54% of asymptomatic adults over the age of 60 [6,7].

The global economic and physical burden of MSK conditions warrants further investigation. Driven largely by an aging population and rising obesity rates, MSK diseases represent the highest category of healthcare spending in the United States, costing approximately $380.9 billion annually in direct costs [8]. Specifically, low back and neck pain account for the largest share of healthcare spending at approximately $134.5 billion, with other MSK disorders totaling $129.8 billion in 2016 [9]. Despite these staggering domestic costs, unequal global treatment increases strain on low- and middle-income countries with limited surgical infrastructure [10]. For instance, there exists 1–1.3 operating theaters per 100,000 population in sub-Saharan Africa and South Asia, compared to 14–25 in North America and Europe. The scale and diversity of MSK disease justify investment in next-generation repair approaches that go beyond what existing surgical approaches offer.

### 1.2. Limitations of Conventional Surgical and Graft-Based Treatments

Surgeons managing MSK defects have relied on graft-based solutions as the primary tool for reconstruction. Existing interventions of conventional grafts and implants address structural failure but not tissue regeneration. Autologous bone graft, most harvested from the iliac crest, remains the gold standard for bridging critical-size defects [11]. However, prospective data demonstrate persistent pain in approximately 2% of patients, and graft availability is finite, limiting use in large defects. Allografts address graft supply limitations but introduce their own distinct concerns. Long-term data demonstrate nonunion rates up to 16%, along with mechanical complications of fracture and reconstruction failure. Variable osteoinductive potential and the risk of disease transmission further complicate their use [12].

For end-stage joint disease, total joint arthroplasty (TJA) provides significant relief but remains a mechanical substitution rather than a biological cure. The 10-year cumulative revision risk ranges from 1.6% to 13%, with younger age categories having a higher risk [13]. Primary failure modes depend on joint and patient factors and include infection, aseptic loosening, and implant wear. Addressing localized chondral defects remains challenging because articular cartilage has a poor intrinsic capacity for healing. While the objective is regenerating organized hyaline cartilage, common bone marrow stimulation techniques are insufficient. Autologous chondrocyte implantation (ACI) results in hyaline-like cartilage rather than fibrocartilage in the defect, which presumably leads to better long-term outcomes and a longer-lasting healing tissue [14]. Soft tissue interventions face similar regenerative hurdles. Tendon repair, even when technically successful, frequently results in scar tissue characterized by inferior collagen alignment and a reduced elastic modulus [15]. Re-rupture rates after Achilles repair range from approximately 0.6% to 6.2%, and rotator cuff re-tears occur in 15% to 21% of cases, depending on tear size and tissue quality [15,16].

### 1.3. Rationale for Bioengineering and Regenerative Approaches

**Figure 1:**
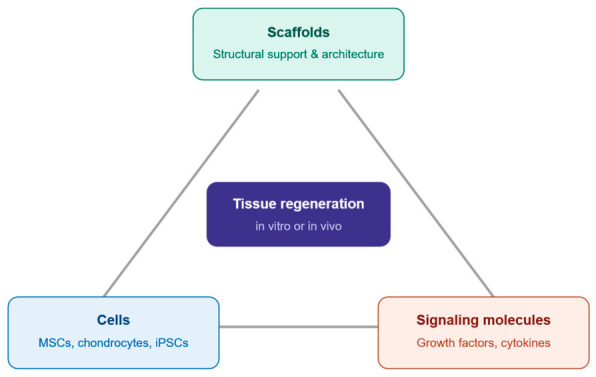
The three-part framework of tissue engineering, integrating scaffolds, cells, and signaling molecules to achieve musculoskeletal tissue regeneration.

Bioengineering approaches aim to replace damaged tissue with living tissue rather than non-living substitutes. The foundation of tissue engineering relies on a three-part framework combining scaffolds, cells, and signaling molecules to regenerate tissues in vitro or in vivo, a concept that has guided the field for decades [17,18] (Figure 1). The core bioengineering objective for MSK repair is to recapitulate the biomimetic functionality and complex hierarchical structure of native tissues. Bone requires a vascularized mineral-organic composite. Cartilage requires a zone-specific extracellular matrix with differential proteoglycan and collagen organization. Tendon requires aligned type I collagen exhibiting specific fiber crimp and mechanical anisotropy. Furthermore, biological fixation requires stratified scaffolds with distinct, continuous tissue regions and gradient mechanical properties [19].

The bioengineering and regeneration field has matured from purely in vitro proof-of-concept studies to a robust pipeline. This includes preclinical large-animal models, early-phase clinical trials, and several Federal Drug Administration (FDA) cleared or approved products, but a substantial translational gap remains. Adoption barriers include a lack of standardization and validation, insufficient physiological complexity, scalability challenges, and regulatory uncertainty. Consistent clinical benefit has not been uniformly achieved, largely because traditional two-dimensional cell cultures and animal models are ineffective at predicting human safety and efficacy [20].

### 1.4. Purpose and Scope of This Review

Despite decades of rapid growth in the field of biomaterials and tissue engineering, the substantial global burden of MSK diseases and its rise outpace the clinical availability of regenerative therapies. Conventional surgical interventions, while adept at addressing gross structural failures, lack inherent regenerative capacity. This review critically assesses the translational feasibility of current scaffold-based systems, cell-based and acellular regenerative platforms, and smart or functionalized materials. These topics span therapeutic paradigms across bone, cartilage, tendon, and ligament repair. We further examine the structural barriers to clinical adoption, including regulatory classification, manufacturing scalability, reimbursement, and access equity. Where existing reviews have focused primarily on biological performance, this review weights translational feasibility as an equally important criterion. Emerging modalities including gene and nucleic acid-based therapies, bioactive peptide-functionalized materials, immunomodulatory biomaterials, and combination scaffold-biologic systems represent active areas of investigation that fall outside the defined scope of this review.

While recent reviews have addressed bone tissue engineering challenges, natural polymer biofabrication and mechanotransduction, regenerative techniques in orthopedic surgery, and tissue engineering for knee cartilage repair, the present review differs in scope and emphasis [21,22,23,24]. Where prior reviews focused on individual tissue types, specific material classes, or single anatomical sites, this review integrates scaffold design, cell-based and acellular approaches, and smart materials across bone, cartilage, tendon, and joint repair within a unified framework. Where existing reviews have treated regulatory pathways, manufacturing scalability, reimbursement, and access equity as secondary considerations, this review positions them as determinative of whether a therapy reaches patients.

## 2. Bioengineering Strategies for Bone and Joint Repair

### 2.1. Biomaterials and Scaffold Design

#### 2.1.1. Natural Versus Synthetic Scaffolds

Scaffold design represents the foundational challenge in MSK tissue engineering, as the material must simultaneously satisfy biological, mechanical, and degradation requirements specific to each target tissue. Natural polymers contain superior properties for cell interaction and biological function, including similarity to native extracellular matrix (ECM), enzymatic degradation pathways, biocompatible degradation products, and high chemical versatility [25]. Among these, decellularized ECM (dECM) is distinguished by its broad composition of matrix proteins, including collagen, elastin, fibronectin, laminin, glycosaminoglycans, and proteoglycans, which provide tissue-specific biochemical cues. These materials have favorable microenvironments that reinforce cellular interactions. However, the mechanical properties of dECM-based constructs alone may not meet the tissue-specific requirements for load-bearing applications [26]. Conversely, synthetic polymers such as polylactic-co-glycolic acid (PLGA), polycaprolactone (PCL), and polylactic acid (PLA) offer avenues for bone regeneration and healing. Although these materials have adequate mechanical strength, they still have limitations, as they release acidic degradation products and exhibit suboptimal cell-material interactions [27].

Scaffolds that integrate a hybrid approach, combining the mechanical advantages of synthetic polymers with the biological functionality of ECM derivatives, represent a promising strategy to overcome the limitations of each material class [28]. These constructs are currently in active preclinical development, with select systems such as PCL-decellularized cartilage ECM (dECM) nanofiber composites and PLGA-cartilage decellularized matrix scaffolds showing favorable results in cartilage regeneration studies [29]. However, few have reached clinical translation (Table 1).

#### 2.1.2. Porosity, Biodegradability, and Mechanical Matching

It is crucial to consider the porosity, biodegradability, and mechanical properties that are tailored to each tissue target. Any mismatches in these factors may contribute to suboptimal results and a performance gap between promising preclinical results and poor clinical outcomes.

Pore size may influence cell behavior and tissue integration. A diameter of approximately 100 µm is the minimum required to accommodate cell size, migration, and nutrient transport [30]. A pore size of 300 µm is recommended for bone applications, as larger pores enhance capillary growth, which is essential for vascularization and, in turn, increases bone formation. Avascular scaffolds such as those used in cartilage repair have distinct requirements [31].

Degradation kinetics must align with the timeline for tissue regeneration. Considering bone reconstruction, the scaffold’s structural integrity should be maintained for at least 1 year before complete in vivo degradation [32]. This is because bone remodeling occurs over extended periods. Conventional preclinical study designs of 3 to 6 months may inaccurately measure the long-term performance of scaffolds, contributing to failures in clinical practice. MSK tissues exhibit varying mechanical properties, and these must be matched appropriately. Articular cartilage contains considerably low Young’s modulus values of 0.80 ± 0.33 MPa for humeral, 0.57 ± 0.17 MPa for patellar, and 0.31 ± 0.18 MPa for femoral cartilage [33]. The patellar tendon, by contrast, exhibits Young’s modulus values of 597 ± 49 MPa, yielding an almost 1000-fold difference that scaffolds must replicate [34].

Beyond structural support, substrate stiffness directly affects stem cell fate decisions. Bone marrow mesenchymal stem cells (MSCs) cultured on stiff substrates (147.4 ± 11.04 kPa) showed increased spreading, proliferation, and osteogenic differentiation, as evidenced by upregulated alkaline phosphatase expression and calcium nodule formation. Conversely, soft substrates (32.73 ± 3.74 kPa) fail to promote osteogenesis, supporting the notion that mechanical cues are not only structural requirements but also active biological signals guiding specific tissue regeneration [35].

#### 2.1.3. Applications Across Bone, Cartilage, and Tendon Interfaces

These material-level design requirements become considerably more complex when scaffolds must span or reconstruct the boundaries between distinct tissue types, where abrupt changes in composition and mechanics demand spatially organized solutions. Interface tissue engineering is among the most clinically significant yet technically challenging aspects of MSK regeneration. Native tissue transition zones depend on organized structural gradients to transfer mechanical loads and distribute stress between tissues with differing properties. Homogeneous, single-material scaffolds cannot replicate this complexity, and these critical junctions are unlikely to achieve functional repair [36].

The osteochondral junction perfectly conveys the sophistication of biological interfaces. Articular cartilage anchors to the underlying subchondral bone through a thin calcified cartilage layer approximately 20 to 250 µm long [36]. This transitional zone contains perpendicularly oriented type II collagen fibers derived from chondrocytes that interlock with type I collagen osteoid produced by osteoblasts. This forms a mechanically strong bond that may withstand repetitive compressive and shear forces without breaking down. Understanding this complexity, osteochondral defect scaffolds have evolved toward gradient-based architectures [37]. Continuous gradients eliminate abrupt interfaces between layers, offering a compelling advantage by reducing stress concentrations and replicating the more seamless transitions inherent to native osteochondral tissue.

Similar principles govern the tendon-bone enthesis, where the Achilles tendon attaches through a specialized 500 µm interface organized into four different zones: tendon, non-mineralized fibrocartilage, mineralized fibrocartilage, and bone. This architecture decreases stress during muscle contraction and prevents failure at an inherently vulnerable junction. Replicating this zonal complexity in engineered scaffolds remains a critical challenge for rotator cuff, anterior cruciate ligament, and Achilles tendon repair [38].

### 2.2. Advanced Fabrication Techniques

#### 2.2.1. 3D Printing and Bioprinting

Replicating the zonal complexity of these interfaces requires fabrication methods capable of producing spatially controlled, multi-material constructs with precision that conventional manufacturing cannot achieve. Additive manufacturing has transformed scaffold fabrication by enabling intricate control over geometry, pore architecture, material composition, and mechanical properties. Acellular metal constructs have already been used in clinical practice. Three-dimensionally printed titanium and cobalt-chrome lattice structures are currently used in spinal interbody fusion devices, acetabular cups, and custom craniofacial implants [39]. This demonstrates the feasibility of additive manufacturing in real-world orthopedic applications. Using these methods, along with computed tomography (CT) or magnetic resonance imaging (MRI), allowed implant designs to be matched to the exact dimensions of an individual patient’s bone defects. These additive manufacturing approaches are separated into three categories: extrusion-based, powder-based, and liquid-based methods [40].

Using hydrogel-based bioinks and integrating living cells into scaffold structures through bioprinting extends these approaches. Common bioinks include alginate, PCL, gelatin, fibrin, collagen, and methacrylated gelatin (GelMA) [41,42]. For bioinks to be effective, they must be printable for scaffold construction while also providing a microenvironment helpful for cell survival. Conventional hydrogels show mechanical weakness due to heterogeneously crosslinked networks and limited energy dissipation. It is also important to note that bioprinting techniques differ in terms of resolution, speed, and cell viability. Inkjet bioprinting offers high resolution (10–80 µm) but moderate viability (74–85%). Extrusion-based printing offers faster scaffold fabrication with varying viability (40–90%), but at reduced resolution, while digital light processing (DLP) achieves ultra-high resolution, high efficiency, and favorable viability (75–95%) [43].

#### 2.2.2. Composite and Gradient Scaffolds

Many MSK tissues have variations in structure, composition, and mechanical properties that require more than just uniform scaffold materials. As a result, scaffold design has shifted toward composite and gradient-based architectures, in which properties such as geometry, porosity, density, and stiffness vary throughout the construct [44]. These three-dimensional scaffolds more closely resemble the heterogeneous organization of the extracellular matrix, enabling engineers to create localized microenvironments that support different cellular behaviors within a single implant. Gradient architectures can also improve the performance of engineered tissues by enhancing nutrient diffusion, cell infiltration, and overall structural stability. One example is in osteochondral repair models, where multi-domain hydrogel constructs have been engineered with stiffness that increases along the scaffold, transitioning from cartilage-like regions (~20 kPa) to bone-like regions (~300 kPa) [45]. The transition layer helps maintain mechanical continuity between phases while reducing the risk of a failed construct and preserving chondrogenic and osteogenic tissue formation.

In addition to compositional gradients, anisotropic structural organization plays a crucial role in the mechanical loading of certain tissues. Tendons and ligaments have collagen fibers that can efficiently transmit tensile forces. Electrospinning generates aligned nanofiber scaffolds that replicate this organization. When comparing randomly oriented fiber networks, the aligned nanofiber sheets possess significantly higher mechanical stiffness, with reported increases of over 100% in some systems [46]. Additionally, a mechanical range can be observed, like that seen in native ligament tissues. These design strategies highlight how spatially controlled architecture is essential for reproducing the structural and functional complexity of MSK tissues.

#### 2.2.3. Vascularization as a Scaffold Design Requirement

Structural and compositional sophistication alone, however, is insufficient if the construct cannot sustain cell viability throughout its volume. This limitation makes vascularization an equally critical design requirement. Vascularization is important for successful MSK tissue engineering and requires scaffold designs that support not only structural integrity but also the transport of oxygen, nutrients, and metabolic waste products. One obstacle to creating engineered tissues is the limited ability of diffusion alone to sustain cells within thick constructs. In most systems, nutrient transport is effective only within approximately 200 µm of a vascular source, limiting the viability of cells farther from it. Beyond its role in cell behavior and tissue integration, scaffold porosity also serves a critical transport function, improving oxygen and nutrient diffusion throughout the construct and reducing hypoxic conditions in thicker implants [47].

Beyond optimizing passive transport, ways to encourage vascular development are increasingly incorporated into scaffold design. Revascularization approaches focus on establishing a microvascular network within engineered tissues before implantation, thereby reducing the time a construct remains avascular and vulnerable to hypoxic conditions. In vitro methods often involve endothelial cell seeding, multicellular spheroid assembly, or cell-sheet technologies to form capillary-like structures within the scaffold matrix. Alternatively, some in situ approaches rely on the host environment to stimulate vascular growth after implantation through angiogenic ingrowth or surgical techniques such as arteriovenous loop models [48].

The general native tissue structures provide important guidance for these design strategies. Within the periosteum, there is a rich blood supply and a population of osteogenic progenitor cells that contribute to bone maintenance and repair. Tissue-engineered periosteal constructs that combine endothelial cells with bone marrow-derived mesenchymal stem cells have been shown to recapitulate aspects of this vascularized approach, supporting improved bone regeneration in experimental models [49]. Together, these findings emphasize that effective scaffold systems must integrate both structural and vascular features to support long-term tissue viability.

## 3. Cell-Based and Acellular Regenerative Approaches

### 3.1. Stem Cell-Based Therapies

Beyond scaffold design, the biological component of tissue engineering relies on the selection and delivery of regenerative cell populations. Stem cell-derived therapies are a major focus point within MSK regenerative medicine because of their capacity for differentiation, immunomodulation, and trophic signaling. Among available cell sources, mesenchymal stem cells, induced pluripotent stem cells (iPSCs), and differentiated chondrocytes are among the most widely investigated for orthopedic applications. Although each approach offers distinct advantages, their clinical effectiveness varies widely.

#### 3.1.1. MSCs, iPSCs, and Chondrocytes

Mesenchymal stem cells are one of the most extensively studied and clinically mature cell populations for MSK regeneration. MSCs can be isolated from multiple tissue sources, including bone marrow, adipose tissue, and perinatal tissues such as umbilical cord and Wharton’s jelly [50]. However, the number of recoverable cells varies widely across these sources. The highest numbers of autologous MSCs per unit volume are found in adipose tissue, with reported ranges of approximately 4700 to more than 1.5 million cells per milliliter of tissue. On the other hand, bone marrow typically produces lower amounts, ranging from roughly 1 to 317,000 cells per milliliter, whereas umbilical cord-derived tissues can yield even higher concentrations of allogeneic MSCs, depending on the collection method. By releasing cytokines, growth factors, and extracellular vesicles, MSCs can stimulate endogenous repair processes, react to inflammation, and promote angiogenesis within damaged tissue. This shift from a direct cell-replacement approach to a paracrine signaling model has fundamentally changed how MSC-based therapies are viewed in regenerative medicine [51].

The most established clinically validated cell-based therapies in orthopedic practice are autologous chondrocyte implantation and its matrix-associated variant. These techniques involve harvesting autologous chondrocytes, expanding them in vitro, and re-implanting them within cartilage defects. During testing, meaningful improvements have been observed. In the SUMMIT trial comparing matrix-induced autologous chondrocyte implantation (MACI) with microfracture for knee cartilage defects, patients receiving MACI showed significantly greater improvements in the Knee Injury and Osteoarthritis Outcome Score (KOOS) pain and function subscores at 2 years [52]. In addition, long-term follow-up studies have further supported these approaches. For those who underwent MACI for knee chondral defects, results at a minimum of 10 years after showed sustained improvements in patient-reported outcomes, successful regeneration on MRI, and generally low rates of reoperation or progression to total knee arthroplasty [53].

Despite these successes, there are still limitations for the broader use of cell-based therapies. There are variable outcomes due to the different cell sources, delivery techniques, and manufacturing methods. Additionally, the survival and persistence of transplanted cells within hostile inflammatory environments remain significant challenges. These limitations have prompted interest in alternative cell sources, including induced pluripotent stem cells. These cells are derived from reprogramming somatic cells into pluripotent states, which enables them to differentiate into a wider array of cell types. These cells may also have increased proliferative capacity and enhanced differentiation potential compared with adult tissue-derived MSCs [54]. Together these factors suggest that iPSC-derived cells could overcome some limitations associated with aging or senescent cell populations.

However, several major barriers currently limit the use of iPSC-based therapies in clinical orthopedics. There are safety concerns, such as the risk of teratoma following implantation [55]. Additionally, immune responses to allogeneic iPSC-derived cells and the complexity of manufacturing clinical-grade cell products present further challenges. As a result, widespread clinical implementation of iPSCs is likely to require substantial advances in safety validation, manufacturing scalability, and regulatory approval pathways, although it has promising future applications in MSK medicine.

#### 3.1.2. Delivery Strategies and Survival Challenges

The therapeutic effectiveness of potent regenerative cells depends on how they are delivered and their retention on the target site. A major obstacle in cell-based orthopedic therapies is the low survival rate of transplanted cells following their implantation [56] (Table 2).

### 3.2. Extracellular Vesicle and Secretome-Based Therapies

#### 3.2.1. Rationale for Acellular Approaches

Acellular regenerative approaches have gained interest in the context of stem cell therapies. Rather than transplanting living cells, these strategies aim to deliver the bioactive factors responsible for regenerative signaling to the target area. Among these factors, extracellular vesicles (EVs), such as exosomes and microvesicles, have emerged as particularly promising therapeutic candidates [59].

Extracellular vesicles are membrane-bound particles released by many cell types, including MSCs. They carry a diverse cargo of proteins, lipids, messenger RNAs, and microRNAs, allowing EVs to modulate numerous biological processes in recipient cells. MSC-derived EVs have been shown to promote angiogenesis, regulate inflammation, and activate multiple regenerative signaling pathways, including PI3K/Akt, Wnt/β-catenin, TGF-β/Smad, and NF-κB pathways [60]. Through these pathways, EVs stimulate osteogenesis, chondrogenesis, and tendon regeneration in preclinical models.

Compared to live cell therapies, acellular approaches offer many advantages. There are logistical and regulatory challenges when transplanting viable cells that EV-based treatments avoid, including issues related to cell survival, tumorigenicity, and immune compatibility. Additionally, EV products may be easier to standardize, store, and distribute, making them appealing options for scalable therapeutic platforms. Platelet-rich plasma (PRP) is among the earliest acellular biologic therapies used clinically in orthopedics. PRP contains concentrated platelets that release growth factors involved in tissue repair and inflammation. Clinical studies suggest that PRP can improve function and reduce pain in conditions such as patellar tendinopathy, knee osteoarthritis, and lateral epicondylitis. However, substantial heterogeneity in preparation methods, platelet concentrations, and cellular content complicates the interpretation of PRP outcomes. This lack of standardized formulations has contributed to inconsistent clinical results and limits support for the method’s efficacy [61].

#### 3.2.2. Evidence from Preclinical and Early Clinical Studies

The growing understanding and preclinical evidence supporting EV-based therapies in MSK repair are continuously expanding. Animal studies across multiple models have demonstrated improvements in bone regeneration, cartilage repair, and soft tissue healing following treatment with MSC-derived EVs. EV-treated groups have shown significant increases in bone volume fraction and new bone formation compared with untreated controls in some experimental bone-healing models. Mechanistic studies suggest that EV-mediated activation of pathways such as mTOR/AKT, AMPK, and bone morphogenetic protein (BMP) signaling contributes to these regenerative effects [62].

Along with these encouraging results, however, limitations in the available clinical evidence remain. While biologic treatments such as PRP have been widely studied in clinical trials, other systematic reviews frequently highlight limitations in methodology, including small sample sizes, heterogeneous treatment protocols, and a high risk of bias during treatment [63]. Although many studies report improvements in pain and functional outcomes, the overall evidence base remains weak. With expanding efforts beyond these limitations, extracellular vesicles may represent the next phase of biologic therapy development. Unlike PRP, which contains a heterogeneous mixture of cellular and plasma components, EV-based therapies offer the potential for more precisely defined molecular products. However, rigorous clinical trials will be required to establish their safety, dosing parameters, and long-term efficacy in orthopedic indications.

Beyond their native cargo, EVs can be deliberately engineered to enhance therapeutic potency through modification of the donor cell environment prior to secretion. Biological preconditioning represents one such approach: culturing MSCs under hypoxic conditions shifts the miRNA profile of secreted EVs, correlating with improved chondrocyte proliferation, reduced apoptosis, and superior cartilage repair outcomes in preclinical OA models compared to normoxia-derived EVs [58]. Physico-chemical methods offer a complementary route. Nanoelectroporation of adipose-derived MSCs has been shown to boost EV yield and load therapeutic mRNAs into secreted vesicles, with the resulting constructs supporting bone regeneration in preclinical defect models [58,64]. Together, these strategies suggest that the therapeutic ceiling for EV-based repair may be considerably higher than what native, unmodified vesicles can achieve (Table 3).

#### 3.2.3. Manufacturing and Standardization Challenges

One of the most significant barriers to the clinical translation of EV-based therapies is the lack of standardized approaches for vesicle isolation, characterization, and potency testing. There are many techniques available for EV separation, including ultracentrifugation, precipitation methods, size-exclusion chromatography, and tangential flow filtration. Each approach differs in efficiency, purity, and scalability, which increases variability in experimental results [65].

Traditional ultracentrifugation methods, widely used, may yield relatively low amounts and can co-isolate contaminating proteins or lipoproteins [66]. Alternative techniques such as tangential flow filtration and size-exclusion chromatography have demonstrated improved scalability and purity. This makes them more attractive for large-scale production. There is still a strong need for universally accepted protocols to improve consistency in findings across studies. Responses to these variable methods have prompted international guidelines, such as the Minimal Information for Studies of Extracellular Vesicles (MISEV) framework, which has been proposed to improve consistency in EV research. Robust quality control requires multi-parameter characterization including particle size distribution, protein marker quantification, and functional in vitro assays relevant to the intended mechanism of action, with these metrics prospectively defined as release criteria before clinical use [67]. These recommendations emphasize the importance of standardized characterization methods, including the use of multiple EV markers and complementary analytical techniques to verify vesicle identity and purity.

Even with improved characterization methods, defining reliable potency assays for EV-based therapeutics remains difficult [68]. The mechanisms of action underlying EV-mediated regeneration are also complex and may vary depending on the disease context, cell source, and vesicle composition. Developing robust assays that accurately predict therapeutic activity, therefore, remains a major challenge for the field. While extracellular vesicles represent one of the most promising emerging techniques in regenerative medicine, much work remains to establish a scalable manufacturing process, standardized quality control metrics, and clinically validated potency assays that can support regulatory approval and widespread clinical adoption.

Advanced Therapy Medicinal Products (ATMPs), governed by European Union Regulation (EC) No 1394/2007, represent a regulatory category of direct relevance to the cell-based and acellular approaches discussed in this section [69,70]. Tissue-engineered products such as MACI, allogeneic MSC therapies, and EV-based constructs may qualify as ATMPs when intended to regenerate or repair tissue, subjecting them to centralized EMA authorization rather than standard device pathways. GMP compliance is required throughout production, including cell sourcing, expansion, and quality release testing, imposing substantially higher manufacturing burdens than conventional device or biologic frameworks. In the United States, analogous products are regulated as biologics under FDA jurisdiction, though classification and pathway requirements differ and early regulatory consultation is advisable for any combination construct [71].

## 4. Smart and Functionalized Materials

### 4.1. Growth Factor Delivery Systems

Beyond biological agents, the material systems used to deliver and support regenerative therapies have themselves become increasingly sophisticated. Growth factor delivery is one of the most consequential and most difficult variables to control in bioengineered MSK repair. Bone morphogenetic protein-2 (BMP-2) is the most clinically established osteoinductive growth factor in the field, with FDA approval for spinal fusion and tibial nonunion [72]. However, its clinical translation has been complicated by a well-documented adverse event burden: independent review of industry-sponsored spine fusion trials found complication rates of 10% to 50% depending on surgical approach, including radiculitis, ectopic bone formation, osteolysis, and urogenital complications [72]. The standard delivery method, a collagen sponge, releases BMP-2 in a high initial burst followed by rapid decline, often requiring supraphysiological dosing to sustain the regenerative signal [73]. However, engineering the release strategy rather than escalating the dose has proven to be the more productive approach for optimizing BMP-2 efficacy [73]. A hyaluronan hydrogel producing low burst followed by sustained release induced up to 456% more bone than a dose-matched collagen sponge, establishing that delivery kinetics are as consequential as growth factor selection [73].

Multi-factor systems have extended this principle through temporally coordinated release. A chitosan-based composite hydrogel released platelet-derived growth factor (PDGF) before BMP-2 in sequence, leveraging PDGF’s early role in recruiting progenitor cells to the repair site before BMP-2’s osteogenic and angiogenic activity begins [74]. Simultaneous delivery of vascular endothelial growth factor, PDGF, and low-dose BMP-2 (2.5 µg) from a single hydrogel system achieved approximately 60% of intact bone mechanics over 28 days, an outcome previously achieved only with high-dose BMP-2 (10 µg) alone [75]. As the field advances, the engineering of delivery kinetics and growth factor combinations offers a path toward equivalent regenerative efficacy with substantially reduced dose-dependent risk [75].

### 4.2. Surface Modifications and Bioactive Coatings

While growth factor delivery addresses the biological signaling environment, implant surfaces present a parallel engineering challenge encompassing both infection resistance and osseointegration. Periprosthetic joint infection (PJI) is a complication of 2.1% of hip and 2.3% of knee arthroplasties in the United States, carries a 20% to 30% five-year mortality rate, and persists as a risk across the lifetime of the implant [76]. Porous additively manufactured implants, increasingly used to promote osseointegration, present a specific vulnerability: their expanded surface area also provides greater opportunity for bacterial colonization [77]. Consequently, building antibacterial activity into the implant surface at the design stage is a more reliable defense than relying on perioperative antibiotics alone [77]. The main surface engineering strategies fall into those that release a bactericidal agent and those that kill on contact, with a third emerging category based on nitric oxide donation. Silver coatings, which release ions that disrupt bacterial membranes and inhibit DNA replication, on trabecular titanium reduced bacterial growth and biofilm formation with minimal host cell cytotoxicity [78]. Clinical data from a systematic review and meta-analysis showed a trend toward reduced PJI risk with silver-coated endoprostheses in both primary oncologic reconstruction and revision cases, though neither result reached statistical significance [79]. Antimicrobial peptides offer a contact-killing alternative: Dhvar5 and MSI78 coatings on titanium were bactericidal against methicillin-resistant Staphylococcus aureus (MRSA) on contact while simultaneously promoting osteoblastic differentiation, suggesting a single surface modification could address both infection resistance and osseointegration [80]. A third strategy relies on covalently linked nitric oxide-releasing groups on polyurethane surfaces, achieving up to 99.99% antibiofilm activity against broad-spectrum bacteria and near-complete MRSA reduction in a murine implantation model [81].

Beyond infection resistance, surface modifications can also actively direct cell behavior. Most synthetic scaffold materials, such as PLGA, PCL, titanium alloys, and hydroxyapatite composites, are biologically inert by default. Without instructive biochemical cues at the surface, cells have limited capacity to adhere, differentiate, or deposit matrix in a controlled fashion. To address this, peptide functionalization converts an inert substrate into one that actively directs cell behavior without the complications of incorporating full-length growth factors [82]. The most established adhesion-promoting sequence is the arginine-glycine-aspartate (RGD) motif, which engages integrin receptors on mesenchymal stem cells to promote adhesion and downstream signaling [82]. Beyond RGD-based strategies, collagen-mimetic peptides extend this principle through a distinct mechanism: the collagen I-derived peptides DGEA and P15 sequences stimulate osteoblastic differentiation directly, upregulating osteocalcin secretion and alkaline phosphatase activity from adherent mesenchymal stem cells and increasing bone formation on hydroxyapatite tibial implants in vivo [83].

## 5. Overview of the Clinical Landscape

The material and biological strategies described above exist within a broader clinical context that varies considerably across anatomical sites and stages of translational maturity. Orthopedic injuries are highly prevalent and impose substantial functional, psychological, and economic burdens. Additionally, many MSK injuries involve tissues with limited intrinsic healing capacity and vascularization, such as cartilage and intra-articular ligaments, which contribute to poor long-term outcomes when left untreated or treated incorrectly. Although biomaterials and tissue engineering strategies have expanded the therapeutic landscape, clinical translation remains uneven, with regenerative efficacy often falling short of native tissue repair and widespread implementation constrained by cost, scalability, and biological complexity.

**Figure 2:**
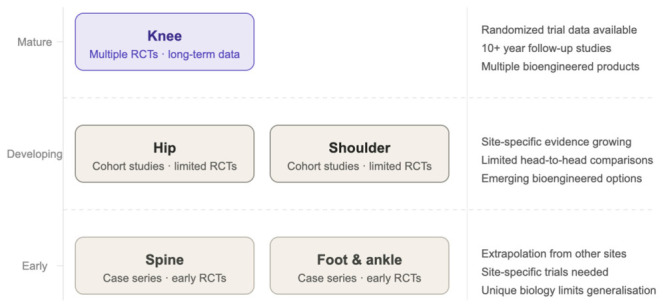
Clinical evidence maturity of bioengineered therapies across musculoskeletal anatomical sites.

Importantly, the quality of evidence and stage of translation vary considerably across anatomical sites and therapeutic approaches (Figure 2). Distinct biological and biomechanical environments across MSK tissues ensure that interventions developed in one context (e.g., the knee) cannot be generalized without independent validation. Differences in joint loading, tissue organization, and healing responses necessitate tailored strategies for each joint. Accordingly, this review presents each anatomical site on its own terms.

### 5.1. Knee: The Reference Standard for Clinical Evidence

#### 5.1.1. Focal Chondral and Osteochondral Defects

The knee represents the most mature clinical landscape for bioengineered cartilage repair, with an extensive comparative evidence base. Currently, MACI serves as the benchmark for the repair of full-thickness cartilage defects. It is supported by randomized data from the SUMMIT trial, demonstrating superior improvements in pain, function, and repair tissue quality compared to microfracture treatment, along with lower failure rates after 2 years [52]. However, these advantages come at a cost, as MACI is a two-stage process involving cartilage harvest, ex vivo expansion, and implantation.

To address cost and scalability concerns with MACI, one-stage scaffold approaches such as autologous matrix-induced chondrogenesis (AMIC^®^) are being explored [84]. This approach has shown promising short-to-mid-term improvement for patients, but comparisons between MACI and AMIC^®^ remain controversial within the field. Outcomes are consistently similar between the two approaches, with systematic reviews unclear on whether one approach provides superior outcomes over the other [85,86]. Ultimately, the variability in tissue qualities, chondral defects, and hospital factors makes this comparison difficult. For larger full-thickness defects, MACI remains the standard of care in many practices due to its established effectiveness. Future clinical trials should continue to explore the difference in outcomes between these two procedures, given the superior cost and one-stage nature of AMIC^®^. Additional trials should focus on modern advancements in chondral repair, such as hyaluronan-based scaffolds and future generations of autologous chondrocyte implants [87].

#### 5.1.2. Knee Osteoarthritis and Intra-Articular Biologics

Intra-articular biologic therapies for knee osteoarthritis represent a predominantly studied application of orthobiotics, yet clinical benefits remain modest and inconsistent [88]. For example, one emergent therapy is mesenchymal stromal cell injections. Despite strong preclinical rationale, including hydrogel delivery systems, these injections have not yet demonstrated clinically meaningful improvements in pain in aggregate analyses [88,89]. A recent meta-analysis of randomized trials found little to no difference in pain at 12 months compared to controls, with effect sizes below the threshold for minimal clinical importance and only low-certainty evidence for functional improvement [88]. Substantial heterogeneity across studies further limits interpretability and highlights the absence of standardized protocols or clear patient selection criteria.

PRP has shown comparatively more favorable outcomes than MSC injections, with meta-analyses demonstrating greater improvements in pain and function relative to hyaluronic acid; however, these effects vary across PRP formulations [90]. Some findings suggest that leukocyte-poor PRP may be associated with better functional outcomes than leukocyte-rich PRP, though the authors noted this finding requires confirmation from direct head-to-head trials, and subsequent RCTs have not consistently replicated this advantage. Further research should investigate the comparative efficacy of PRP and corticosteroids, as current evidence does not clearly establish superiority of either approach.

#### 5.1.3. Meniscus and Ligament Reconstruction

Meniscus scaffold replacement represents a well-established bioengineered application in ligamentous and meniscal pathology, with a growing clinical evidence base supporting its use in symptomatic post-meniscectomy patients. Systematic review and meta-analysis data demonstrate that both collagen meniscus implants (CMI) and polyurethane-based (Actifit) scaffolds yield significant improvements in patient-reported outcomes and activity levels, with no meaningful differences between implant types [91]. Reported failure rates remain relatively low, and imaging studies suggest acceptable scaffold integration and tissue ingrowth over time. These findings position meniscal scaffolds as a viable joint-preserving option, particularly in younger populations.

In contrast, biological augmentation of ligament reconstruction remains largely investigational. While extensive preclinical work supports strategies targeting graft incorporation and ligamentization, including growth factors, stem cells, and biomaterials, clinical evidence is limited and heterogeneous [92]. Existing studies report mixed outcomes and are frequently constrained by suboptimal methodology and inconsistent endpoints. As a result, despite strong mechanistic rationale, bioengineered approaches to ligament healing have not yet achieved the same level of clinical translation as meniscal scaffold applications.

### 5.2. Hip and Total Joint Applications

#### 5.2.1. Hip Cartilage Repair and Joint Preservation

While the knee provides the most mature evidence base, emerging data suggest that bioengineered interventions can delay or even avoid total hip arthroplasty (THA) in younger patients, achieving meaningful outcomes in anatomically and biomechanically distinct environments [93,94,95]. In one study on patients with femoroacetabular impingement, autologous matrix-induced chondrogenesis (AMIC) produced sustained improvements in modified Harris Hip Scores over 8 years, whereas conventional microfracture outcomes deteriorated after one year, with 22% of microfracture patients requiring THA [94]. Injectable autologous chondrocyte implantation adapted to the hip’s spherical geometry shows promising results, with high rates of defect fill and significant improvements in patient-reported outcomes at two-year follow-up, highlighting the feasibility of two-stage, cell-based interventions in this challenging anatomical site [95].

Osteonecrosis of the femoral head represents another target for joint-preserving biologic strategies. Core decompression augmented with bone marrow-derived stem cells improves pain and function in this region, reduces disease progression, and lowers the rate of conversion to THA compared with core decompression alone [93]. Bioengineered scaffolds may improve the standard of care for hip pathologies, especially for patient populations that would benefit from a motion-preserving alternative to THA. However, trial depth and long-term comparative evidence remain limited compared with those for the knee.

#### 5.2.2. Periprosthetic Applications in Total Joint Arthroplasty

Bioengineering strategies for periprosthetic bone loss, infection prevention, and revision fixation are highly relevant to arthroplasty practice. Periprosthetic bone mineral density loss is common after total joint arthroplasty and increases the risk of implant loosening and fracture, with contributors including stress shielding, surgical technique, patient age, and gaps in postoperative management [96]. Pharmacologic interventions, including anabolic agents, antiresorptives, and other compounds, show efficacy in preserving periprosthetic bone, and local delivery via bioactive coatings further enhances osseointegration while mitigating systemic side effects [97]. For bone reconstruction in revision arthroplasty, bioengineered scaffolds such as tricalcium phosphate/hydroxyapatite graft substitutes are increasingly used as autograft extenders, achieving long-term survival comparable to allografts in challenging acetabular defects [98].

Periprosthetic infection remains a significant clinical challenge, with 1–2% of arthroplasties affected and high associated mortality [77]. Antibacterial implant coatings, including the silver-based technologies discussed above, are being actively applied in the periprosthetic setting to reduce infection risk without compromising bone healing. Additionally, these coatings can be paired with patient-specific 3D-printed titanium implants, which demonstrate promising short- and mid-term outcomes in severe acetabular defects that are not compatible with conventional implants, demonstrating improved functional scores and implant stability [99].

### 5.3. Shoulder: Rotator Cuff and Glenohumeral Cartilage

#### 5.3.1. Rotator Cuff Repair Augmentation

Similar joint-preservation principles extend to the shoulder, where bioengineering strategies address both soft tissue failure and articular cartilage loss in a mechanically distinct environment. Rotator cuff repair augmentation with extracellular matrix or synthetic patches is supported by strong clinical evidence, specifically indicated in cases of large tears with poor tissue quality. Re-tear rates for the rotator cuff increase sharply with size, ranging from 10% for small tears to over 50% for tears greater than 6–8 cm^2^, making structural augmentation an important consideration in higher-risk cases [100,101]. Clinical studies demonstrate that patch augmentation improves repair integrity, with healing rates of 60–85% compared with roughly 40% for nonaugmented repairs, and is associated with modest improvements in range of motion and functional scores [101]. Allograft patches appear to provide the greatest improvements in postoperative pain and external rotation, although complication rates are slightly higher than standard repair, and rigorous clinical evaluation remains limited [100].

In contrast, PRP augmentation has not shown a clinically meaningful functional benefit in rotator cuff repair. While some studies report lower structural re-tear rates with PRP compared to the standard of care, improvements in patient-reported outcomes and pain scores are not statistically significant or clinically meaningful [102].

#### 5.3.2. Glenohumeral Cartilage and Shoulder Arthroplasty Context

The shoulder connects bioengineering with arthroplasty through techniques such as biologic glenoid resurfacing and management of periprosthetic bone loss. Articular cartilage defects in the glenohumeral joint are uncommon, typically occurring after trauma, recurrent instability, or prior surgery, and are often discovered incidentally during other procedures [103]. Consequently, the evidence base for cartilage repair in the shoulder is limited compared with the knee. Midterm outcomes for hemiarthroplasty with biologic glenoid resurfacing using human dermal matrix allograft are encouraging, with most patients reporting high satisfaction, acceptable functional scores, and low revision rates, demonstrating the potential of biologic strategies to preserve joint function in select patients, but further research is needed to expand the currently small evidence pool [104].

Periprosthetic shoulder applications similarly benefit from bioengineering approaches. Innovative biomaterials such as bioactive glass and tantalum are being used to enhance osseointegration, fill bone defects, and deliver local antibiotics in the setting of periprosthetic joint infection or severe humeral bone loss [105]. Long-term data on glenoid components indicate that cemented polyethylene implants achieve markedly higher survival than cementless metal-backed designs, demonstrating the importance of material selection for joint preservation [106].

### 5.4. Spine

Beyond peripheral joints, spine surgery offers perhaps the most instructive example of both the promise and the pitfalls of translating bioengineered therapies into widespread clinical practice. Recombinant human bone morphogenetic protein-2 (rhBMP-2) achieved widespread adoption following FDA approval as an autograft substitute for spinal fusion, becoming one of the most extensively studied biologics in the field [107]. However, subsequent independent analyses revealed higher-than-reported complication rates with off-label use and dose escalation of rhBMP-2 [72]. Importantly, rhBMP-2 has not demonstrated clear superiority over autograft and may be associated with additional risks, such as malignancy [108]. Alongside rhBMP-2, demineralized bone matrix (DBM) and calcium phosphate ceramics are currently used as graft extenders, integrating bioengineering into contemporary spinal fusion practice [109]. These technologies provide context for emerging regenerative strategies in the spine, particularly intervertebral disk repair. While substantial preclinical progress has been made with polymers, growth factors, and cell-based therapies, clinical translation remains limited for many of these technologies. Safe and effective adoption is critical to the future of bioengineering in spine surgery.

### 5.5. Foot and Ankle

#### 5.5.1. Unique Biomechanical and Biological Context

In contrast to the spine, where high surgical volumes have accelerated clinical adoption, the foot and ankle present a distinct set of biological and biomechanical challenges that have slowed translational progress. Joint loading in the ankle can reach up to four times body weight during routine ambulation, placing substantial stress on repair constructs and regenerated tissue [110]. At the same time, key structures such as the talus have a limited vascular supply and extensive cartilage coverage, which limit intrinsic healing capacity and increase vulnerability to complications following injury [111]. Soft tissue considerations further complicate management, as local tissue damage is a strong predictor of adverse outcomes, often outweighing systemic comorbidities in determining treatment success [112].

Patient-specific biological factors also play a critical role in outcomes, specifically in high-risk populations. For example, patients with complicated diabetes and peripheral neuropathy have markedly increased risks of surgical site infection, emphasizing the importance of patient selection [113]. Additionally, osteochondral lesions of the ankle have variable outcomes, largely dependent on lesion size, depth, containment, and patient characteristics [114]. Overall, the foot and ankle cannot be approached through extrapolation from knee or hip results and require site-specific evidence to integrate bioengineering principles into the standard of care.

#### 5.5.2. Talar Osteochondral Defects

Talar osteochondral defects represent the most active area of bioengineered cartilage repair in the foot and ankle, addressing a clear unmet clinical need. These lesions commonly arise following ankle trauma in patients aged 20–40 and frequently present with persistent pain and dysfunction [115]. Conventional marrow stimulation techniques such as microfracture are effective for small lesions but demonstrate diminishing returns beyond established size thresholds, with poorer outcomes in defects greater than 1.5 cm [115]. As a result, scaffold-based and cell-based approaches are increasingly utilized for larger or more complex lesions where traditional strategies are insufficient.

Emerging clinical evidence supports the potential of these approaches to improve outcomes. AMIC has demonstrated significant pain reduction, high rates of defect fill, and successful return to sport at mid-term follow-up [116]. In randomized comparisons, scaffold augmentation strategies such as BST-CarGel combined with microfracture have shown superior improvements in pain, function, and radiographic healing compared to microfracture alone or microfracture with PRP [117]. High-quality trials should continue to define optimal indications and long-term durability of these technologies.

#### 5.5.3. Achilles and Foot Tendon Augmentation

The Achilles tendon augmentation literature provides a clear and instructive example that biologic augmentation must be evaluated on a product-specific, evidence-based basis. High-level evidence demonstrates no meaningful benefit for commonly used augmentation strategies for the Achilles [118,119]. A meta-analysis found that ECM scaffold augmentation did not improve patient satisfaction, rupture rates, or infection outcomes compared with standard repair, while a large multicenter PATH-2 randomized trial showed that PRP injections conferred no improvement in tendon function, patient-reported outcomes, or quality of life. This gap between theoretical rationale and clinical outcomes highlights the limitations of extrapolating theoretical benefits into clinical efficacy. The Achilles tendon literature reinforces that not all products within a class demonstrate meaningful benefit, despite promising results seen in knee and hip applications.

#### 5.5.4. Bone Repair and Reconstruction in the Foot and Ankle

Bone repair in the foot and ankle spans a broad clinical spectrum, from routine fusion augmentation to complex reconstruction of the talus, with outcomes often dictated more by patient biology than by scaffold technology alone. A wide array of bone graft substitutes, such as calcium sulfate, calcium phosphate, tricalcium phosphate, bioactive glass, and demineralized bone matrix, are already well established as autograft extenders in fusion procedures [120]. Clinical evidence supports the use of growth factor-augmented constructs such as rhPDGF-BB/β-TCP, which demonstrate fusion rates comparable to autograft with reduced pain and donor-site morbidity [121,122]. Similarly, adjunctive use of rhBMP-2 in high-risk patients has shown high union rates and acceptable safety profiles, although usually in off-label contexts [123].

At the more complex end of the spectrum, reconstruction of severe talar pathology highlights both the potential and limitations of current approaches. While fusion procedures can achieve high rates of osseous union, they are associated with substantial complication rates, particularly in medically complex populations [124]. Emerging solutions such as patient-specific talar body prostheses demonstrate improved functional outcomes and long-term survivorship compared to arthrodesis in selected patients, offering a joint-preserving alternative in otherwise devastating pathology [125]. Risk stratification, careful patient selection, and evaluation of all available options are critical to optimizing outcomes in foot and ankle reconstruction.

### 5.6. Cross-Site Synthesis: What the Anatomical Comparison Reveals

Despite site-specific differences in biology, loading, and evidence maturity, the translational challenges across anatomical sites are all related to heterogeneous study populations, limited high-level evidence, and insufficient long-term follow-up (Table 4). Across sports medicine and reconstructive literature, many studies remain low-level evidence, often lacking a control group [126]. Implant use is most reported in the knee, followed by the shoulder and ankle, but across all sites, the literature is characterized by small cohorts and limited comparative follow-up [126]. Broader reviews of regenerative therapies reinforce that outcomes are highly dependent on local biological environments, with variable results driven by factors such as vascularity, mechanical loading, and insertion-site biology [127]. These findings demonstrate that despite widespread adoption of biologic and bioactive implants, robust comparative evidence remains insufficient to establish them as a standard of care.

A unifying theme across joint systems is the convergence of biological repair strategies with arthroplasty science. TJA failures are primarily driven by aseptic loosening and infection, prompting parallel development of functional coatings designed to enhance osseointegration while preventing bacterial colonization [128]. This convergence supports a framework in which arthroplasty and repair are not competing paradigms but complementary approaches within a shared strategy. Coordinated, cross-site evidence generation with multidisciplinary trial design represents the future of bioengineering in orthopedics.

## 6. Translational Challenges in Clinical Adoption

### 6.1. Regulatory and Manufacturing Considerations

The anatomical heterogeneity and evidence gaps identified above reflect deeper structural barriers that impede the translation of bioengineered therapies across all sites. Regulatory classification of orthopedic products into devices, biologics, and combination products is a fundamental determinant of clinical translation pathways and the evidence requirements set by governing bodies. In the United States, most orthopedic devices (>97%) are cleared through the 510(k) pathway, while higher-risk devices require premarket approval with full clinical validation, and biologics follow a separate license application pathway. Acellular scaffolds functioning primarily as structural supports are generally classified as Class II devices eligible for 510(k) clearance, whereas scaffolds incorporating cells or growth factors are regulated as combination products subject to premarket approval [129]. Combination products introduce additional regulatory complexity because they integrate device, drug, and/or biologic components under a single therapeutic strategy. For novel combinations, full International Council for Harmonisation (ICH) compliant toxicology programs may be required for each component, along with additional combination-specific studies, significantly increasing cost and development timelines [130]. Alternative frameworks, such as the European Union’s hospital exemption pathway, allow limited, non-routine clinical use of advanced medicinal products for individual patients under national oversight, which enhances access but raises concerns about variable standards of evidence and safety [131]. In contrast, standard orthopedic devices in the European Union are regulated under the Medical Device Regulation (European Union MDR 2017/745), with CE marking granted through Notified Body conformity assessment rather than FDA review. EN ISO 13485 [132] provides a harmonized manufacturing quality standard recognized across both systems [133,134]. Biocompatibility testing under ISO 10993 is required for all implantable constructs, with test selection determined by contact duration and tissue type; degradable scaffolds require additional evaluation of degradation products [135].

Scalable, reproducible manufacturing is a primary determinant of whether bioengineered therapies successfully reach clinical practice. Standardized closed-system bioreactors are one such scalable process that can generate billions of MSCs with improved consistency compared with traditional planar culture systems, but substantial challenges remain in quality control across batches [136]. These limitations are even more pronounced in advanced biologics, such as extracellular vesicle-based therapies, where defining identity, purity, sterility, potency, and stability is unreliable and batch-to-batch consistency is limited [68]. Patient-specific 3D-printed implants represent a distinct field in which early identification systems have been established. Quality by Design approaches emphasize early identification of critical quality attributes and integration of risk management across design and manufacturing for these additively manufactured implants [137]. However, this framework requires deep multidisciplinary coordination and regulatory confidence that may be difficult to adapt to quality assessments for other products.

### 6.2. Economic and Implementation Barriers

The high per-patient cost of bioengineered therapies reflects the inherent complexity of biologically active, precision-manufactured products and the infrastructure required to produce them. Cell-based therapies such as MACI incur substantial costs driven by two-stage procedures, cell expansion, and stringent manufacturing and handling requirements, resulting in markedly higher total costs compared to single-stage alternatives [138]. Advanced implant technologies, such as patient-specific implants, further compound these expenses by requiring high-resolution imaging, computer-aided design, and sophisticated manufacturing processes, such as 3D printing with extensive post-processing and quality assurance [139]. Even at the developmental stage, manufacturing costs are substantial, with facility and personnel requirements representing most of total development expenditures, and these are layered on top of the significant regulatory burden associated with clinical trials and approval pathways for high-risk therapies [129,140]. These factors create a cost structure that limits accessibility and scalability for broader clinical adoption of novel therapeutics.

Beyond manufacturing costs, reimbursement uncertainty represents a critical late-stage barrier to the successful translation of bioengineered therapies, as regulatory approval does not guarantee payer coverage or patient access. Analyses of novel technologies show that fewer than half achieve Medicare coverage, often taking several years post-approval, with only a small minority gaining reimbursement within the first few years [141]. This reflects a fundamental mismatch between regulatory and payer standards: while the FDA evaluates safety and efficacy, payers such as Centers for Medicare and Medicaid Services (CMS) require evidence that interventions are “reasonable and necessary,” often incorporating cost-effectiveness and long-term durability, which are not required for FDA approval [142,143]. As a result, products with limited long-term data or evolving techniques, such as autologous chondrocyte implantation platforms, have faced delayed or restricted coverage despite regulatory authorization [144,145]. Compounding this, approved regenerative therapies may fail commercially without reimbursement, as developers often prioritize regulatory approval over payer alignment. Cost-effectiveness assessments are highly sensitive to assumptions about durability and comparative outcomes; many products lacking robust longitudinal data are hampered by payer hesitancy. Early integration of health economic modeling into development pipelines is therefore critical to ensuring payer alignment alongside regulatory approval.

**Figure 3:**
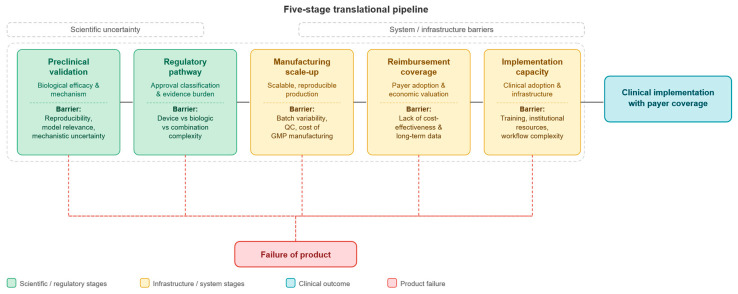
Translational barriers pipeline. Schematic representation of the sequential stages required for clinical translation of bioengineered orthopedic therapies. Each stage is associated with a dominant barrier, and product failure can occur at any stage. Stages shift from primarily scientific challenges to structural constraints in later stages, marked by a shift in color from green (scientific) to orange (infrastructure), with brown representing a mixture of both.

Even when reimbursement is secured, learning curves and institutional requirements represent additional structural barriers to the equitable adoption of bioengineered therapies. Many bioengineering procedures, such as autologous chondrocyte implantation, require mastery of multi-stage workflows that demand specialized surgical skills and multidisciplinary infrastructure beyond standard orthopedic training [146,147]. Successful implementation depends not only on operative technique but also on institutional capacity for cell processing, quality control, and coordinated postoperative care [147]. These requirements create a concentration of expertise in high-resource centers, limiting broader dissemination. More broadly, disparities in surgical infrastructure, especially in community, rural, and low-resource centers, mean that access to advanced bioengineered interventions is often constrained by institutional capability rather than patient need or insurance status [148]. Addressing these infrastructure disparities requires deliberate investment rather than relying on product availability to drive adoption. Figure 3 diagrams the sequential barriers that products encounter, from preclinical validity to implementation capacity.

## 7. Access and Equity Considerations in Bioengineered Therapies

### 7.1. Influence of Cost and Infrastructure on Availability

The structural barriers described above do not affect all patients equally. Cost, infrastructure, and institutional concentration create systematic disparities in who can access bioengineered therapies. Advanced regenerative therapies are disproportionately available in high-income settings, and the gap is growing. A study of 10 representative biotherapeutic products across 40 countries found that affordability is projected to worsen by 1.5 to 2 times in upper- and lower-middle-income countries by 2030 and by 4 to 6 times by 2040, while remaining essentially stable in high-income countries [149]. Cost alone does not explain this disparity. Access to cell and gene therapies is also constrained by infrastructure limitations, regulatory hurdles, and shortages of specialized personnel and stem cell processing capacity, barriers that pricing reforms cannot resolve on their own [150]. These patterns are visible within domestic orthopedic care. A national survey found that 32% of orthopedic practices limited access for adult Medicaid patients, with private practices showing higher restriction rates than academic ones, and simulated Medicaid patients were significantly less likely to be offered timely appointments than those with commercial insurance [151]. For advanced cell and gene therapies specifically, the payment structure compounds this disparity: the high upfront cost of single-dose products conflicts directly with Medicaid’s fixed budget model designed around chronic care expenditure [152]. Manufacturing requirements add a further layer of constraint that exists independent of cost or insurance. Producing induced pluripotent stem cell-based therapeutic products under Good Manufacturing Practice (GMP) conditions requires reprogramming, characterization, banking, and scaled expansion, a process that demands specialized infrastructure and expertise and frequently prevents clinical trials from launching [153].

### 7.2. Concentration in Specialized Centers and Geographic Inequity

The geographic distribution of orthopedic surgical expertise in the United States has become progressively more concentrated in urban centers. The share of the orthopedic workforce located in metropolitan counties grew from 77% in 2000 to 93% in 2019, indicating that existing workforce trends are deepening rather than correcting this imbalance [154]. The clinical consequences for rural patients are significant. Rural orthopedic care is challenged by delayed presentation, underestimation of urgency, subspecialty service deficits, and deficiencies in local support, with providers specifically noting that treatment options requiring frequent travel should be avoided [155]. Multi-stage procedures such as matrix-associated autologous chondrocyte implantation, which require an initial biopsy followed by six weeks of cell culture prior to implantation, impose precisely this kind of logistical burden on patients already facing significant barriers to subspecialty access. The cycle perpetuating this shortage is itself structural: limited rural representation among medical students, inadequate residency exposure to rural practice, and increasing sub-specialization of trainees collectively sustain a deficit that rural communities cannot resolve independently [156].

Within the orthopedic workforce, advanced cartilage restoration procedures are increasingly concentrated among fellowship-trained subspecialists. From 2003 to 2015, cartilage restoration procedures declined significantly among recently trained orthopedic surgeons, with the sharpest decline in marrow stimulation techniques, suggesting that procedural expertise is not being broadly taught during residency training [157]. Even where subspecialty surgeons are present, the broader surgical infrastructure required to support advanced procedures is frequently absent in rural settings: 55.1% of rural counties had no surgeon of any kind, 81.2% had no anesthesiologist, and 58.1% had no certified registered nurse anesthetist [158]. For bioengineered procedures that additionally require specialized cell processing, advanced imaging, and trained rehabilitation professionals, these systemic gaps further compound existing access barriers.

### 7.3. Design Choices Are Access Determinants

Therapy design directly determines who can access a treatment, often more than efficacy does. PRP and bone marrow aspirate concentrate (BMAC) can be prepared intraoperatively from a patient’s own blood or bone marrow using commercially available centrifuge systems, requiring no specialized manufacturing infrastructure or cold-chain logistics (temperature-controlled transport and storage) [159]. MACI, by contrast, requires GMP-certified manufacturing, extensive quality control, and cold-chain transport between two procedures separated by approximately six weeks; these demands effectively confine it to high-volume academic centers [160]. The regulatory pathway, determined at the design stage, is therefore a primary determinant of geographic reach independent of clinical efficacy [160]. This accessibility advantage comes at a cost: the heterogeneity in PRP and BMAC preparations across commercially available systems reflects the tension between accessibility and standardization. The potential for standardizing bioengineered therapy without sacrificing design simplicity remains an unresolved challenge for the field [161]. Resolving this tension will require deliberate consideration of access implications at the earliest stages of therapy design, rather than treating equity as a downstream implementation problem.

## 8. Emerging Directions and Future Opportunities

### 8.1. Design for Manufacturability and Scalability from Inception

Addressing the translational, economic, and equity barriers identified above will require fundamental shifts in how bioengineered therapies are designed, evaluated, and deployed. Despite notable advancements in bioengineered MSK therapies, their clinical translation remains constrained, partly because manufacturability is inadequately considered during the initial stages of development. Historically, research efforts have predominantly focused on biological performance, often neglecting the economic, regulatory, and production constraints that ultimately influence clinical adoption. Successful translation necessitates a “translational mindset,” wherein scalability, manufacturability, and regulatory feasibility are integrated into the design process from the outset [162].

Evidence from regenerative medicine underscores the repercussions of failing to adopt this approach. Therapies that secure regulatory approval do not necessarily achieve reimbursement or widespread utilization, demonstrating a disconnect between development priorities and real-world implementation [144]. Furthermore, traditional manufacturing methods, particularly manual, open-system cell processing, are costly, labor-intensive, and prone to contamination, thereby limiting reproducibility and scalability.

Future endeavors should therefore prioritize simplified, scalable manufacturing strategies. Promising directions include the development of allogeneic cell therapies that facilitate standardized, large-scale production, as well as acellular approaches such as extracellular vesicle-based therapies, which may offer enhanced stability and storage through techniques like lyophilization [163,164]. Advances in automated and closed-system manufacturing platforms, including bioreactors and isolator-based systems, further demonstrate the potential to mitigate contamination risk, enhance consistency, and reduce production costs [165]. Together, these approaches reflect a necessary paradigm shift in the field: designing therapies not only for biological efficacy but also for practical, scalable implementation. Without this shift, even the most promising bioengineered solutions may fail to reach patients.

### 8.2. Integration of Real-World Evidence into Translational Pipelines

Beyond manufacturing considerations, the translational pipeline also requires better integration of clinical outcomes data. While preclinical models have provided valuable mechanistic insights, their capacity to predict clinical success remains limited. Small-animal models, including rodent systems, exhibit thinner cartilage and greater intrinsic healing capacity than humans, potentially leading to overestimation of therapeutic efficacy. Even larger animal models, such as canine or porcine systems, fail to fully replicate human joint size and cartilage structure, further limiting their translational relevance [166].

These limitations underscore the need to complement preclinical findings with robust real-world clinical data. Orthopedic registries offer a powerful mechanism to capture outcomes across diverse patient populations and practice settings, enabling evaluation of procedure utilization, complication rates, and comparative effectiveness that is not feasible in controlled trials [167]. Importantly, registries provide longitudinal, population-level data that reflect the variability inherent in clinical care, thereby improving the generalizability of findings.

Future efforts should prioritize the development of standardized clinical registries and biorepository-linked data systems to support post-market surveillance and rigorous evaluation of biologic therapies. Consensus recommendations emphasize that institutions offering these treatments should commit to structured data collection frameworks that enable consistent outcome reporting and cross-institutional comparison [168]. These approaches are essential for establishing the clinical credibility of bioengineered therapies, informing reimbursement decisions, and supporting the development of evidence-based guidelines necessary for widespread adoption.

### 8.3. Data-Driven Design and Personalized Approaches

Alongside registry-based evidence, computational approaches offer a complementary path toward more precise and efficient therapy development. Emerging computational and data-driven methodologies hold the potential to fundamentally transform the design and implementation of bioengineered therapies. Traditionally, scaffold development has relied on iterative, trial-and-error experimentation across an extensive, multidimensional design space, making optimization both time-consuming and costly. Computational modeling frameworks now facilitate in silico exploration of design parameters, enabling researchers to systematically assess combinations of material and structural features and predict resultant graft performance prior to experimental validation [169].

Recent advancements in high-throughput screening, coupled with machine learning, further enhance this capability by enabling the rapid evaluation of multiple scaffold architectures and the identification of optimal structural parameters. By integrating nonlinear modeling approaches, these methods can clarify complex relationships between scaffold features, such as porosity, surface area, and permeability, and biological outcomes, including osteogenic gene expression [170].

Beyond material design, analogous data-driven strategies are being applied to patient stratification. Multivariable models incorporating imaging and biochemical biomarkers, ranging from MRI-derived cartilage metrics to serum and urine markers, have demonstrated the capacity to predict disease progression with moderate accuracy, underscoring both the promise and data requirements of personalized approaches [171]. These tools are genuinely promising, but the gap between proof-of-concept and clinical utility is worth stating plainly. Predictive models built on single-institution datasets frequently fail to generalize; the biomarker signatures that predict OA progression in one cohort perform modestly in another. The same problem applies to scaffold optimization: a machine learning model trained on one biomaterial family may not generalize to another. Progress here is less a matter of algorithmic sophistication and more a matter of data infrastructure, specifically large, harmonized, prospectively collected datasets that simply do not yet exist at the scale these methods require. The relative difficulty of assembling such datasets, compared with developing the models themselves, helps explain why proof-of-concept studies continue to outpace clinical implementation in this area [172]. Collectively, these advancements support a transition from population-based design toward precision orthopedics, where therapies are optimized not only for material performance but also for individual patient biology, ultimately enhancing outcomes and facilitating more efficient clinical translation.

### 8.4. Strengths and Limitations of This Review

This review differs from much of the existing literature by treating translational feasibility as a criterion of equal weight to biological performance. Spanning scaffold design, cell-based therapies, acellular approaches, and smart materials offer a broader vantage point than reviews focused on a single tissue type or technology. This review pays explicit attention to regulatory classification, manufacturing scalability, reimbursement, and global access equity, addressing gaps that more biology-focused reviews tend to leave open.

The narrative approach carries real limitations. Without predefined search criteria or systematic inclusion protocols, the review may miss some relevant work. Existing clinical evidence is also uneven across anatomical sites, as the knee is supported by robust randomized trial data, while many other anatomical sites only have case series or early cohort studies, making cross-site comparisons difficult. Finally, given the rapid advancements in this field, some technologies described here will have moved on by the time of publication.

## 9. Conclusions

Bioengineered bone and joint repair have advanced considerably over the past two decades, progressing from early proof-of-concept studies to a clinical pipeline that includes FDA-cleared scaffolds, autologous cell-based therapies, and functionalized implant surfaces. The knee remains the anatomical site with the most robust clinical evidence, while the hip, shoulder, spine, and foot and ankle continue to lag in trial depth and long-term comparative data. Across all sites, however, a consistent pattern emerges: biological complexity cannot be resolved by optimizing any single component in isolation.

Failures in clinical translation have frequently resulted from mismatches between in vitro performance and the mechanical, immunological, and biological demands of the in vivo environment. Scaffold architecture, cell source, growth factor release kinetics, vascularization strategy, and surface bioactivity each contribute independently to outcomes. Conventional small-animal models compound this problem, as species with thinner cartilage and greater intrinsic healing capacity systematically overestimate therapeutic efficacy before human trials.

Several priorities follow from this review. Manufacturing scalability and design simplicity are primary determinants of clinical reach, not secondary commercial considerations. Acellular approaches, including extracellular vesicle-based therapeutics and off-the-shelf scaffold systems, hold promise given their compatibility with standardized production and broader deployment. Regulatory strategy and health economic modeling must be integrated earlier in development, as reimbursement barriers have consistently prevented viable therapies from reaching patients following approval. Access and equity also warrant deliberate attention from the outset, as design decisions made early in development, including procedure staging, cold-chain dependence, and infrastructure requirements, directly determine which patient populations can realistically benefit.

Emerging tools, including computational scaffold optimization, machine learning-assisted patient stratification, and standardized clinical registries, provide a credible framework for more efficient and generalizable evidence generation. Progress in these areas depends less on algorithmic sophistication than on the assembly of large, harmonized, prospectively collected datasets that do not yet exist at the required scale. Translational feasibility must be weighted as a core design criterion from the earliest stages of development, not an implementation problem deferred until after regulatory approval. Addressing the full burden of MSK disease will require sustained coordination across engineering, clinical, regulatory, and health systems disciplines, with that principle applied consistently throughout.

## Figures and Tables

**Table 1:** Scaffold Design Decision Framework: Tradeoffs Between Biological Activity and Mechanical Performance Across Tissue Types and Material Categories.

Tissue Type	Bone	Cartilage	Tendon/Ligament	Osteochondral Interface
**Natural Polymers:** Collagen, Gelatin, Extracellular Matrix (ECM), Chitosan, Hyaluronic Acid	**Pro:** Bioactive, promotes cell adhesion **Con:** Insufficient mechanical strength for load-bearing **Examples:** Collagen-hydroxyapatite composites **Clinical:** Limited standalone use	**Pro:** ECM-mimetic, supports chondrogenesis **Con:** Low mechanical strength, rapid degradation **Examples:** Collagen, chitosan, hyaluronic acid, alginate **Clinical:** Matrix-induced autologous chondrocyte implantation (MACI) uses collagen membrane	**Pro:** Native ECM composition, cell recognition **Con:** Inadequate tensile strength, variable sourcing **Examples:** Collagen, dECM, silk fibroin **Clinical:** dECM grafts available	**Pro:** Supports both chondral and osseous zones **Con:** Cannot achieve mechanical gradient alone **Examples:** Collagen-based bilayers **Clinical:** TruFit (withdrawn), limited options
**Synthetic Polymers:** Polylactic-co-glycolic acid PLGA, PCL, Polylactic Acid	**Pro:** Tunable degradation, adequate strength **Con:** Acidic degradation, poor bioactivity **Examples:** PLGA, PCL **Clinical:** FDA-approved devices exist	**Pro:** Controlled degradation, structural integrity **Con:** Limited cell-material interaction **Examples:** PCL, PLA **Clinical:** Investigational only	**Pro:** High tensile strength, aligned fiber fabrication **Con:** Inflammatory response, limited bioactivity **Examples:** PCL, PLA electrospun fibers **Clinical:** Non-absorbable grafts (e.g., polyester)	**Pro:** Gradient stiffness achievable **Con:** Lacks zonal bioactivity **Examples:** PCL gradient scaffolds **Clinical:** Investigational only
**Ceramics:** Hydroxyapatite, Tricalcium Phosphate, Bioactive Glass, Zirconia	**Pro:** High compressive strength, osteoconductive **Con:** Brittle, slow resorption **Examples:** Hydroxyapatite, tricalcium phosphate, zirconia **Clinical:** Widely used clinically	**Pro:** N/A—ceramics inappropriate for cartilage **Con:** Excessive stiffness, no compliance **Examples:** None **Clinical:** Not used	**Pro:** N/A—ceramics inappropriate for soft tissue **Con:** No flexibility, incompatible mechanics **Examples:** None **Clinical:** Not used	**Pro:** Bone phase support, mineralization **Con:** Cannot support cartilage phase **Examples:** Hydroxyapatite/tricalcium phosphate in bone layer **Clinical:** Used in bone layer only
**Hybrid/Composite Systems**	**Pro:** Balanced mechanics + bioactivity **Con:** Complex manufacturing **Examples:** PCL-hydroxyapatite, PLGA-bioactive glass **Clinical:** Emerging products	**Pro:** Mechanical support + bioactivity **Con:** Interface stress concentrations **Examples:** Gelatin-chitosan-silk, PCL-collagen **Clinical:** Limited clinical products	**Pro:** Strength + biological cues + anisotropy **Con:** Complex alignment requirements **Examples:** PCL-collagen nanofibers, dECM-hyaluronic acid **Clinical:** Investigational	**Pro:** Replicates native gradient architecture **Con:** Manufacturing complexity, regulatory hurdles **Examples:** Collagen-hydroxyapatite gradients, multiphasic constructs **Clinical:** Few products; MaioRegen (Europe)

**Table 2:** Cell delivery strategies and survival outcomes in orthopedic stem cell therapies.

Strategy	Mechanism	Key Finding	Limitation
Direct injection (saline)	Bolus cell delivery to target site	~10% cell retention immediately post-implant	Rapid cell loss to circulation; inflammatory clearance [56]
Biomaterial-assisted delivery (injectable hydrogel)	Encapsulation provides mechanical protection and retention	~50–60% cell retention immediately post-implant	Requires optimization of stiffness and degradation kinetics [57]
Hypoxic preconditioning	Metabolic conditioning before transplantation reduces apoptosis	Improved post-implant survival in animal models	Additional manufacturing step; not yet standardized [58]

**Table 3:** Summary of extracellular vesicle engineering strategies for musculoskeletal repair.

Strategy	Method	Mechanism	MSK Application
Biological preconditioning	Hypoxic culture (5% O_2_) of donor MSCs	Shifts miRNA cargo of secreted EVs	Cartilage repair in OA
Physico-chemical engineering	Nanoelectroporation of donor MSCs	Activates mTORC1-autophagy axis; boosts EV yield and mRNA loading	Bone regeneration [64]
Biomaterial-assisted delivery	Injectable hydrogel encapsulation	Improves EV retention and controlled release at injury site	Bone and soft tissue repair [57]

**Table 4:** Clinical evidence by anatomical site. Summary of bioengineered orthopedic applications stratified by anatomical site. The stage of clinical translation indicates whether a joint has well-established comparative trials for bioengineered technologies; ‘mature’ denotes that a site does, and ‘developing’ denotes that a site still has only early case studies or RCTs. ‘Representative Bioengineered Approaches’ is not an exhaustive list, but rather a summary of the major technologies discussed in this review; similarly, ‘Primary Translational Challenge’ is not exhaustive either for each technology.

Anatomical Site	Knee	Hip	Shoulder	Spine	Foot & Ankle
Representative Bioengineered Approaches	MACI, AMIC, PRP/MSC injections	AMIC, bone marrow-derived stem cell augmentation	ECM patch augmentation, biologic glenoid resurfacing, cemented polyethylene implants	BMP-2, DBM, graft extenders	AMIC, scaffolds, rhPDGF-BB, rhBMP-2, bone graft substitutes
Best Available Clinical Evidence	Multiple Randomized Controlled Trials (RCTs) and meta-analyses	Cohort studies, limited comparative trials	Smaller RCTs, cohort studies	RCTs, long-term observational data, post-market surveillance	Small RCTs, case series
Primary Translational Challenge	Durable tissue quality and patient selection	Fixation, durability, infections	High mechanical load and inconsistent clinical benefits	Dose safety, adverse events	Extreme loading, patient comorbidities, poor cross-translation with other joints
Stage of Clinical Translation	Mature	Developing	Developing	Mature	Developing

## Data Availability

No new data were created or analyzed in this study. Data sharing is not applicable to this article.

## Abbreviations

The following abbreviations are used in this manuscript:

ACI	Autologous Chondrocyte Implantation
AMIC	Autologous Matrix-Induced Chondrogenesis
BMP	Bone Morphogenetic Protein
BMP-2	Bone Morphogenetic Protein-2
BMAC	Bone Marrow Aspirate Concentrate
CMI	Collagen Meniscus Implant
CMS	Centers for Medicare and Medicaid Services
CT	Computed Tomography
DALYs	Disability Adjusted Life Years
DBM	Demineralized Bone Matrix
dECM	Decellularized Extracellular Matrix
DLP	Digital Light Processing
ECM	Extracellular Matrix
EVs	Extracellular Vesicles
FDA	Food and Drug Administration
GBD	Global Burden of Diseases, Injuries, and Risk Factors Study
GelMA	Methacrylated Gelatin
GMP	Good Manufacturing Practice
ICH	International Council for Harmonisation
iPSCs	Induced Pluripotent Stem Cells
KOOS	Knee Injury and Osteoarthritis Outcome Score
MACI	Matrix-Induced Autologous Chondrocyte Implantation
MISEV	Minimal Information for Studies of Extracellular Vesicles
MRI	Magnetic Resonance Imaging
MRSA	Methicillin-Resistant Staphylococcus Aureus
MSCs	Mesenchymal Stem Cells
MSK	Musculoskeletal
OA	Osteoarthritis
ON	Orthoregeneration Network
PCL	Polycaprolactone
PDGF	Platelet-Derived Growth Factor
PJI	Periprosthetic Joint Infection
PLA	Polylactic Acid
PLGA	Polylactic-co-glycolic Acid
PRP	Platelet-Rich Plasma
RCTs	Randomized Controlled Trials
RGD	Arginine-Glycine-Aspartate
rhBMP-2	Recombinant Human Bone Morphogenetic Protein-2
rhPDGF-BB/β-TCP	Recombinant Human Platelet-Derived Growth Factor BB/Beta-Tricalcium Phosphate
THA	Total Hip Arthroplasty
TJA	Total Joint Arthroplasty
